# Specimen collection for electron microscopy.

**DOI:** 10.3201/eid0604.000427

**Published:** 2000

**Authors:** H R Gelderblom, P R Hazelton


5. Chen M, Fan MY, Bi DZ, Zhang JZ. Sequence analysis of PCR products amplified from five strains of spotted fever group Rickettsiae with Rr with Rr 190. 70p and Rr 190. 602n primers. Acta Virol 1998;42:91-3.
6. Zhang JZ, Fan MY, Bi DZ. Isolation and identification of spotted fever group Rickettsiae BJ-93 strain. Chin J Microbiol Immunol 1997;1:28-33.
7. Fournier PE, Roux V, Raoult D. Phylogenetic analysis of spotted fever group Rickettsiae by study of the outer surface protein OmpA. Int J Syst Bacteriol 1998;48:839-49.
8. Roux V, Raoult D. Phylogenetic analysis of the genus *Rickettsia* by 16S rDNA sequencing. Res Microbiol 1995;146:385-96.
9. Roux V, Rydkina E, Eremeeva M, Raoult D. Citrate synthase gene comparison, a new tool for phylogenetic analysis, and its application for the Rickettsiae. J Syst Bacteriol 1997;47:252-61.
10. Stothard DR, Fuerst PA. Evolution analysis of the spotted fever and typhus group of *Rickettsia* using 16S rRNA gene sequences. Syst Appl Microbiol 1995;18:52-61.
11. Weisburg WG, Dobson ME, Samuel JE, Dasch GA, Mallavia LP, Baca O, et al. Phylogenetic diversity of the Rickettsiae. J Bacteriol 1989;171:4202-6.

### Specimen Collection for Electron Microscopy

**To the Editor:** As virologists whose specialties include diagnostic electron microscopy (EM), we read with interest the discussion on bioterrorism scenarios (1,2) and the subsequent note by Marshall and Catton (3) on the rapid EM diagnostic process used for smallpox (1). EM diagnostics for viral agents offer an open, undirected view; a catch-all method; and speed. A negative stain preparation may be made and a result could be obtained within 5 minutes of the specimen's arrival in the EM laboratory. As suggested by Marshall and Catton, however, success depends as much on the quality of the sample collected as on the method of preparation and skill of the microscopist.

The Konsilarlaboratorium für die Elektronenmikroskopische Erregerdiagnostik in the Robert Koch Institut, Berlin, Germany, provides EM viral diagnostic services for up to 800 specimens per year and counsels other German diagnostic units. The Electron Microscope Unit for the Department of Medical Microbiology and Infectious Diseases, University of Manitoba, is used for EM viral diagnostics by both the major health-care facility in Manitoba, Canada, and the Manitoba Provincial Laboratories; it examines approximately 2,300 clinical specimens annually. Our two facilities examine 70 to 90 vesicular specimens of suspected viral origin annually. In our experiences, the most effective methods of specimen collection from virus-induced blisters (or ulcers) involve opening the vesicle with a 26-gauge needle. The exudate may then be collected and prepared for examination in one of three ways: 1) Draw lesion aspirates into the barrel of the needle with a tuberculin syringe and cap the needle (4); 2) touch a light microscope slide to the vesicle fluid; or 3) touch a 400-mesh, plastic-coated specimen grid directly to the base of the lesion (5). The samples may then be transported to an EM facility for preparation and examination. With the first two sample types, the sample is resuspended in approximately 20 µL of 0.2-µ pore-filtered, bidistilled water; this suspension is used to prepare a standard drop preparation on a 400-mesh, carbon-reinforced, plastic-coated grid. In all cases, the specimens are then negatively stained and examined.

Because of safety concerns about HIV infection, many health officials view transport of vesicle aspirates in capillary pipettes or needles as unacceptable. Glass slides are considered more acceptable, but still a risk. Since examination facilities or wards usually do not have the material to do direct touch preparations onto EM grids, many health officials advocate placing samples into transport medium. Alternatively, swabs may be used to prepare smears on glass slides for subsequent EM examination (6). Swabs in transport medium may be of value for culture or polymerase chain reaction procedures. However, in our experience these samples are not acceptable for EM diagnostics. Marshall and Catton suggest skin scrapings as an alternative to swabs (3). We find that these samples are preferable to swab specimens but not ideal. Our success rates in identifying herpesvirus and orthopoxvirus by drop method preparation (7-9) of vesicle aspirates are 62% to 80%, annually. The advent of sample transport as swabs has made additional procedures necessary to improve sensitivity and has delayed results. In Manitoba, direct centrifugation of samples to EM grids with the Beckmann Airfuge (Palo Alto, California, USA) is used as a nonspecific method of concentrating virus in sample preparations. This method increases the yield of viral particles by three or more orders of magnitude (8,10). In spite of this concentration method, the success rate in EM diagnostics using swab specimens has declined to <10%, while viral agents continue to be identified in >60% of lesions in submitted aspirates.

Because concentration methods are not always available, and in view of the sample problems identified by Marshall (3), we reviewed, in Winnipeg, whether collection of lesion fluids directly onto EM sample grids (5) improved sensitivity over aspiration into 26-gauge needles on tuberculin syringes (4). While neither method increased the number of cases identified in matched samples, the yield of virus seen in samples taken by touching the EM sample grid directly to the base of the lesion did increase, making it easier to identify viral agents in the samples (Hazelton and Louie, unpub. data). In Berlin, we also routinely find higher particle numbers on grids that have been prepared by the direct touch method. Sample preparation on EM grids is conducive to prolonged storage and transport of samples over long distances (5) and removes the risk of needle-stick accidents.

We continue to recommend examining grids touched directly to the lesion or vesicle aspirates. Where possible, infectious diseases and infection control staff contact the EM unit when a sample needs to be collected to receive instructions about methods and ensure that staff are available to conduct the examination. When the specimen needs to be transported some distance, such as between cities, smears on individually packaged glass slides or on sample grids are an alternative method for submitting vesicle aspirates. Glass slides allow the collection of samples for both polymerase chain reaction and EM examination (Charles Humphrey, personal communication). An additional advantage of smears is that interfering background proteins can be removed by drying the sample on the slide and then resuspending the viral agent. Proteins such as mucus, which interfere with staining and visualization, remain insoluble. We understand that other major viral EM diagnostic units also prefer aspirates, smears on glass slides, or lesion exudate on the final sample grid as preferred methods of submission of suspected blister material because of ease in handling and higher efficiency in examination.

### Acknowledgment

We thank Charles Humphrey, Tom Louie, and Sara Miller for their observations.

**Hans R. Gelderblom* and Paul R. Hazelton†**
*Konsilarlaboratorium für die
Elektronenmikroskopische Erregerdiagnostik
in the Robert Koch Institut, Electron Microscopy
and Imaging Group, D13353 Berlin, Germany;
and †Electron Microscopy Unit, Department of
Medical Microbiology and Infectious Diseases,
University of Manitoba, Canada

### References

1. O'Toole T. Smallpox: An attack scenario. Emerg Infect Dis 1999;5:540-6.
2. Henderson DA. Smallpox: clinical and epidemiologic features. Emerg Infect Dis 1999; 5:537-9.
3. Marshall JA, Catton MG. Specimen collection for electron microscopy. Emerg Infect Dis 1999;5:842.
4. Burtonboy G, Lachapelle JM, Tennstedt D, Lamy ME. Detection rapide des virus au microscope electronique. Interet de la methode de coloration negative pour le diagnostic de certaines dermatoses d'origine virale. Ann Dermatol Venerol 1978;105:707-12.
5. Van Rooyen CE, Scott MA. Smallpox diagnosis with special reference to electron microscopy. Can J Public Health 1948;39:467-77.
6. Flewett TH. Some recent contributions from the electron microscope laboratories. BMJ 1972;1:667-70.
7. Gelderblom HR, Renz H, Özol M. Negative staining in diagnostic virology. Micron Microscopica Acta 1991;22:435-77.
8. Hammond GW, Hazelton PR, Chuang I, Klisko B. Improved detection of viruses by electron microscopy after direct ultracentrifuge preparation of specimens. J Clin Microbiol 1981;14:210-21.
9. Biel SS, Gelderblom HR. Electron microscopy of viruses. In: Cann A, editor. Virus cell culture—a practical approach. Oxford: Oxford University Press;1999:111-47.
10. Gelderblom H, Reupke H. Rapid viral diagnosis using the Airfuge. IV. (Abstract W50A/9). International Congress on Virology. The Hague; 1976: p. 630.

### Antimicrobial Resistance

**To the Editor:** Davis et al. offered four reasons why local antimicrobial selection pressure in cattle may not play an important role in the dissemination of multidrug-resistant *Salmonella* from cattle to humans (1). Their conclusions differ from those of other recent studies (2-6).

The authors' first two arguments relate to the high levels of chloramphenicol resistance in the United States, despite a relative lack of

